# Engineering of Fatty Acid Synthases (FASs) to Boost the Production of Medium-Chain Fatty Acids (MCFAs) in *Mucor circinelloides*

**DOI:** 10.3390/ijms20030786

**Published:** 2019-02-12

**Authors:** Syed Ammar Hussain, Ahsan Hameed, Md. Ahsanul Kabir Khan, Yao Zhang, Huaiyuan Zhang, Victoriano Garre, Yuanda Song

**Affiliations:** 1 Colin Ratledge Center for Microbial Lipids, School of Agriculture Engineering and Food Science, Shandong University of Technology, Zibo 255049, China; ammarshah88@yahoo.com (S.A.H.); ahsanhameed@outlook.com (A.H.); kabir_khan@sdut.edu.cn (M.A.K.K.); zhangyao@sdut.edu.cn (Y.Z.); zhyuan004@126.com (H.Z.); 2 Department of Biology, South Texas Center of Emerging Infectious Diseases (STCEID), University of Texas, San Antonio, TX 78249, USA; 3 Departamento de Genética y Microbiología (Unidad asociada al IQFR-CSIC), Facultad de Biología, Universidad de Murcia, 30071 Murcia, Spain; vgarre@um.es

**Keywords:** medium-chain fatty acids, metabolic engineering, *Mucor circinelloides*, microbial lipids, bio-fuels

## Abstract

Increasing energy demands and health-related concerns worldwide have motivated researchers to adopt diverse strategies to improve medium-chain fatty acid (MCFA) biosynthesis for use in the functional food and aviation industries. The abundance of naturally produced MCFAs from botanical sources (i.e., coconut fruit/seeds and palm tree) has been observed to be insufficient compared with the various microorganisms used to cope with industrial demands. *Mucor circinelloides* is one of many promising microorganisms; it exhibits diverse biotechnological importance ranging from the production of functional lipids to applications in the manufacture of bio-fuel. Thus, research was conducted to acquire the desired elevated amounts of MCFAs (i.e., C8–C12) from metabolically engineered strains of *M. circinelloides* M65. To achieve this goal, four different acyl-acyl carrier protein (ACP) thioesterase (TE)-encoding genes exhibiting a substrate preference for medium-chain acyl-ACP molecules were expressed in *M. circinelloides* M65, resulting in the generation of C8–C12 fatty acids. Among all the engineered strains, M65-TE-03 and M65-TE-04 demonstrated the highest production of non-native C8–C10 and C12 fatty acids, respectively, in comparison to the control. These recombinant strains biosynthesized MCFAs de novo within the range from 28 to 46% (i.e., 1.14 to 2.77 g/L) of total cell lipids. Moreover, the reduction in chain length eventually resulted in a 1.5–1.75-fold increase in total lipid productivity in the engineered strains. The MCFAs were also found to be integrated into all lipid classes. This work illustrates how the integration of heterologous enzymes in *M. circinelloides* can offer a novel opportunity to edit the fatty acid synthases (FAS) complex, resulting in increased production of microbial MFCAs.

## 1. Introduction

Medium-chain fatty acids (MCFAs) and their derivates with a chain length of 8 to 12 [1] are involved in the production of green fuels. These MCFAs also offer vital roles as promising intermediates for diverse biochemical industries, such as the production of detergents, bio-plasticizers, surfactants, perfumes, adhesives, and substrates for flavor and fragrance [2,3,4,5,6,7,8]. Over the previous decade, these MCFAs have gained attention as part of a healthy diet because they are absorbed and transported directly into the liver via portal veins and rapidly metabolized by the β-oxidation pathway, eventually augmenting diet-induced thermo-genesis. This process offers a platform for the prevention/treatment of diverse metabolic disorders, including atherosclerosis, hypertension, hyperlipidemia, obesity, type-II diabetes, and cardiovascular diseases [9]. The concentration of naturally produced MCFA from botanical sources has been found to be insufficient to fulfill industrial demand [10,11]. Therefore, the current research focus has shifted towards the de novo biosynthesis of MCFAs from different oleaginous microorganisms [10,11,12,13,14,15,16,17,18]. These microorganisms represent a reliable option for the production of fatty-acid-derived products on an industrial scale [19,20,21,22,23].

**Figure 1:**
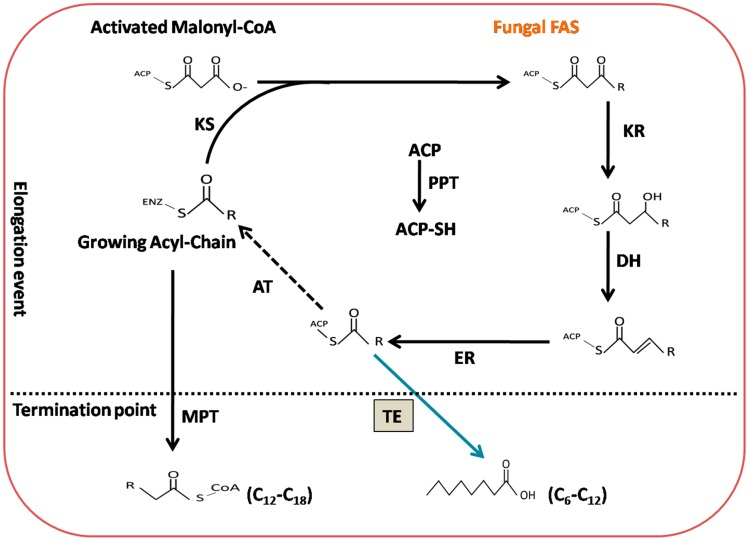
Design and strategy for engineering the fungal fatty acid synthases (FASs) to produce medium-chain fatty acids (MCFAs). Reaction cycles catalyzed by different domains of engineered fungal FASs. A heterologous medium-chain thioesterase (TE) was integrated into FASs (as shown in grey box) to release MCFAs. ER: enoyl reductase; DH: dehydratase; MPT: malonyl/palmitoyl transferase; ACP: acyl carrier protein; KS: ketoacyl synthase; KR: ketoacyl reductase; PPT: phosphopantetheinyl transferase; AT: acetyl transferase.

Over the past two decades, numerous approaches have been adopted in different microorganisms to enhance MCFA content; likewise, the reversal/disruption of β-oxidation pathway engineering in *Saccharomyces cerevisiae* and *Escherichia coli* has been performed [12,14,15,16,17,24], as well as diverse metabolic engineering strategies for the fatty acid synthase (FAS) complex protein in *Yarrowia lipolytica*, *E. coli*, and *S. cerevisiae* by modification of the keto-synthase (KS) and acetyl-transferase (AT)-binding sites, reducing the dimensions of the KS active pockets and ACP-acyl chain binding sites and inducing domain switching of malonyl-palmitoyl transferase (MPT) with thioesterase protein (TE). In addition to using different approaches, most oleaginous microorganisms produce mostly long-chain (16- and 18-carbon) fatty acids (LCFAs), consistent with the preferential activities of transacylases and acyl-ACP-thioesterases towards a longer acyl-AC chain length [24,25,26]. Taken together, the aforementioned investigations have reported low-titer production of MCFAs, despite a great demand for MCFAs in functional foods and in the biochemical and aviation industries [9,10,11,13,18,27,28,29,30,31]. To circumvent the challenges discussed above, there is an immediate need for the selection of an appropriate oleaginous microorganism and suitable strategy/tool. The mechanism responsible for fatty acid biosynthesis by the FAS complex in *M. circinelloides* is depicted in Figure 1. Fatty acid biosynthesis is initiated when acetyl-CoA condenses with carbon dioxide (CO_2_) via acetyl-CoA carboxylase (ACC) to produce malonyl-CoA. The acetyl-CoA and malonyl-CoA subsequently enter the reaction center of FAS via the acetyltransferase activity (AT) to form acetyl-ACP or malonyl-ACP, respectively. These ACPs have been previously activated by phosphopantetheinyl transferase (PPT). The malonyl-ACP then undergoes a decarboxylation event, and ketosynthase (KS) catalyzes its condensation with acetyl-ACP. The remaining domains of the FAS complex include the following: ketoreductase (KR), dehydratase (DH), and enoylreductase (ER) catalysis in a sequential manner to facilitate removal of the oxygen atom and double bonds among carbons to eventually produce a saturated acyl-ACP molecule. Finally, acyl-ACP molecules are available as a substrate for the further addition of decarboxylated malonyl-ACP. In the fungal FAS complex, the elongation step through the addition of malonyl-CoA to the growing acyl-chain continues until malonyl/palmitoyl transacylase (MPT) catalyzes the fatty acid termination step to produce long-chain fatty acids. Although fungal FAS are kinetically more efficient and encompass a distinct configuration, no thioesterase (TE) activity was detected. However, in the bacterial and plant FAS complex, the thioesterase preference is the key metabolic control for the fatty acid chain length [23,32,33,34]. *M. circinelloides* is regarded as a model organism for investigations of lipid accumulation. It has been extensively genetically manipulated for the production of diverse biotechnological precursors for the functional food and bio-fuel industries. However, its fatty acid profile indicates that it is mostly formed by long-chain fatty acids (LCFAs), ultimately making it less attractive for multi-purpose industrial applications [35,36,37,38,39,40,41,42]. Therefore, an attempt was made, for the first time, to integrate the diverse heterologous thioesterase (TE) proteins from bacterial and plant sources into the FAS complex of *M. *circinelloides** strain M65. This approach allows the production of MCFAs in significant titers. In addition, we evaluated the capability of heterologous thioesterases (TEs) to associate with the native FAS complex in *M. circinelloides* M65 [36] and to cease the elongation step prematurely in order to overproduce MCFAs. We revealed that the expression of diverse bacterial and plant acyl-ACP-thioesterases in *M. circinelloides* M65 ultimately generated higher lipid-producing mutants with reduced-chain-length fatty acids.

## 2. Results

### 2.1. Generation of Diverse TE Gene-Over-Expressing Strains by Genetic Manipulation

**Figure 2:**
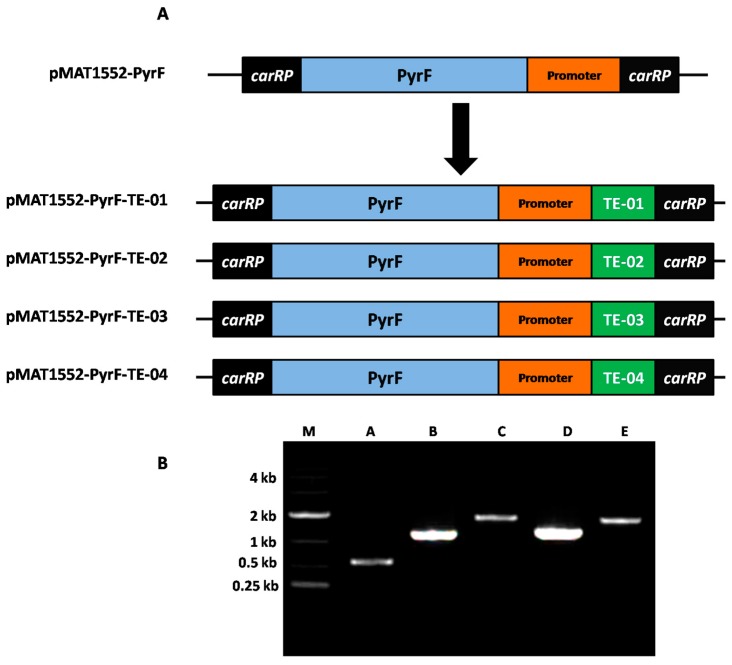
(**A**,**B**) Expression of *TE-01*, *TE-02*, *TE-03*, and *TE-04* genes. (**A**) Structure of plasmids pMAT1552-pyrF-TE-01, pMAT1552-pyrF-TE-02, pMAT1552-pyrF-TE-03, pMAT1552-pyrF-TE-04 for *TE-01*, *TE-02*, *TE-03*, *TE-04* genes over-expressing in *M. circinelloides* M65 are demonstrated. Green boxes indicate the coding region of thioesterase genes. (**B**) Polymerase chain reaction (PCR) amplification of genome of control strain (A) and thioesterase gene (TE) over-expressing strains i.e., M65-TE-01, M65-TE-02, M65-TE-03, and M65-TE-04 was demonstrated to as B–E, respectively with the primers (Supplementary materials: Table S2). M, Gene Ruler DNA Ladder Mix. Sizes in kb of the relevant maker fragments are indicated.

Over-expressing strains of *M. circinellodes* M65 were constructed to evaluate the involvement of different TE genes in lipid accumulation and MCFA overproduction. To achieve our aim, genes *TE-01*, *TE-02*, *TE-03*, and *TE-04* were cloned from the genome of *Anaerococcus tetradius*, *Cuphea palustris*, *Clostridium perfringens,* and *Umbellularia californica*, respectively, and subsequently inserted into the expression vector pMAT1552-pyrF [37], which contains a promoter, the *pyrF* gene, as a selectable marker, flanked by sequences of the TE genes and adjacent regions (Figure 2A) (See Section 4 for details). Transformation and selection for positive colonies were conducted as described by Rodríguez-Frómeta et al. [35]. We obtained 24 transformants from 32 independent transformations (i.e., 8 for each gene). As *Mucor* spores are multi-nucleate in nature and have coenocytic (aseptated) hyphae, vegetative rounds of growths are obligatory to enrich for recombinant nuclei. Three transformants for each gene that stably produced white-colored progeny were selected in comparison to the control strain. Integration of target genes (TEs) into the genome of the resultant transformants was confirmed by polymerase chain reaction (PCR) analysis using primer pairs (Supplementary materials: Table S2). This process consequently amplified the TEs and 542 bp sequence of plasmid pMAT1552-pyrF. The PCR product fragments for each TE transformant were 1241 bp, 1781 bp, 1286 bp, and 1694 bp, as expected, in the corresponding recombinant genomes, whereas a 542 bp fragment was amplified from the control strain M65 (i.e., M65 carrying the empty vector pMAT1552-PyrF) (Figure 2B). The PCR amplification results confirmed that the target gene was integrated into the genome of the over-expressing strains. The PCR results validated the integration of the desired genes (TEs) into the recipient fungal strains (M65). Three clones of each engineered strain (i.e., M65-TE-01, M65-TE-02, M65-TE-03, and M65-TE-04) were grown in complete medium for 4 d in 1-L baffled flasks containing 150 mL of modified Kendrick and Ratledge (K and R) medium, and the lipid contents were calculated. Finally, only the sample with the highest lipid-producing engineered strain for each TE gene was selected for further analysis.

### 2.2. Expression Levels of Different TE Genes in TE-Over-Expressing Strains

Real-time quantitative reverse transcription polymerase chain reaction (qRT-PCR) was conducted to evaluate the mRNA levels of the different acyl-ACP thioesterase genes (i.e., *TE-01*, *TE-02*, *TE-03*, and *TE-04*) in comparison to the control strain (i.e., M65 exhibiting the empty vector pMAT1552-PyrF). All strains (i.e., control and over-expressing strains) were grown in a 3-L fermenter with modified K and R medium, and samples were collected at predefined time intervals of 24, 48, and 72 h. The finding that the mRNA for all TE genes (i.e., *TE-01*, *TE-02*, *TE-03*, and *TE-04*) was maintained at elevated levels throughout the whole culture time eventually validated the expression of TE genes in transformants carrying the respective over-expressing plasmids.

### 2.3. Cell Growth and Lipid Yield from the Engineered Fungal Strains

**Figure 3:**
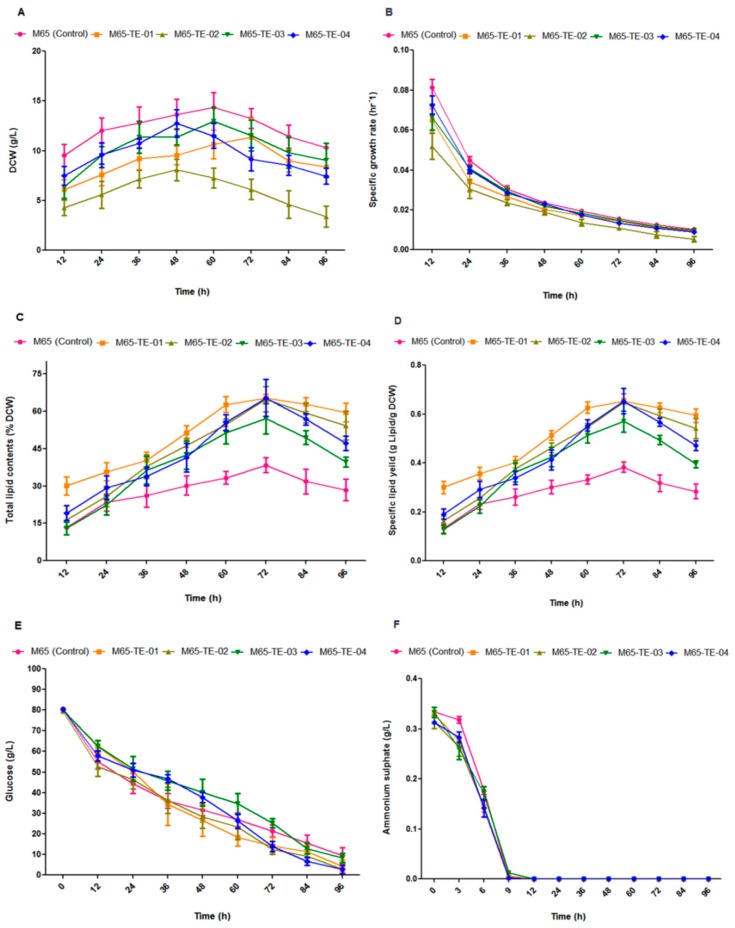
(**A**–**F**) Cell growth, lipid accumulation and substrate consumption of control and engineered strains cultivated in 3-L fermenter with 1.5 L modified K and R medium for 96 h. (**A**) Dry cell weight (DCW), (**B**) specific growth rate, (**C**) percentage of total lipid content from total DCW, (**D**) specific lipid yield, (**E**) residual glucose concentration, (**F**) ammonium sulphate concentration. Values were mean of three independent experiments. Error bars represent the standard error of the mean.

Cell growth, total lipid contents, and the specific lipid yield in all engineered strains (i.e., M65-TE-01, M65-TE-02, M65-TE-03, and M65-TE-04) were compared with the control strain (M65) over 96 h of cultivation in a 3-L fermenter (Figure 3). The growth of all TE-over-expressing strains were inhibited, especially for the M65-TE-02 strain (<50%) from initial to the later growth stage as shown in Figure 3A,B. The dry cell weight (DCW) of all TE over-expressing strains were varies and lower than control from the initial cultivation stage (12 h), indicating the gene expression was effected from the start of the cultivation. In addition, TE over-expression also caused profound shift on the growth stages as most of the engineered strains has a shorter stationary stage except for M65-TE-01, and growth decreased after 72 h of cultivation (Figure 3A,B). This condition might be due to most of the metabolic flux of the TE over-expressing strains were directed to lipid production as the lipid produced by these strain were significantly higher than control (Figure 3A–D). All the engineered strains showed a significant elevation of lipid accumulation ranging from 57 ± 4% to 65 ± 3% whereas the control strain demonstrated a lipid accumulation of approximately 36±2% (Figure 3C). In addition to TLC, the engineered strains, i.e., M65-TE-01, M65-TE-02, M65-TE-03, and M65-TE-04, produced 0.65 ± 0.015, 0.64 ± 0.052, 0.57 ± 0.061, and 0.65 ± 0.074 g lipid per gram DCW, respectively. The control strain (M65) was only able to accumulate 0.36±0.29 g lipid per gram dry cell weight, demonstrating that the improved specific lipid productivity was due to reduced growth of engineered strains leading to enhance de novo fatty acids synthesis (Figure 3D). The pattern of glucose and nitrogen consumption remained almost similar, but comparatively rapid glucose consumption was noticed for strain M65-TE-04 (Figure 3E,F).

### 2.4. Production of De Novo Fatty Acids in M. Circinelloides M65

**Figure 4:**
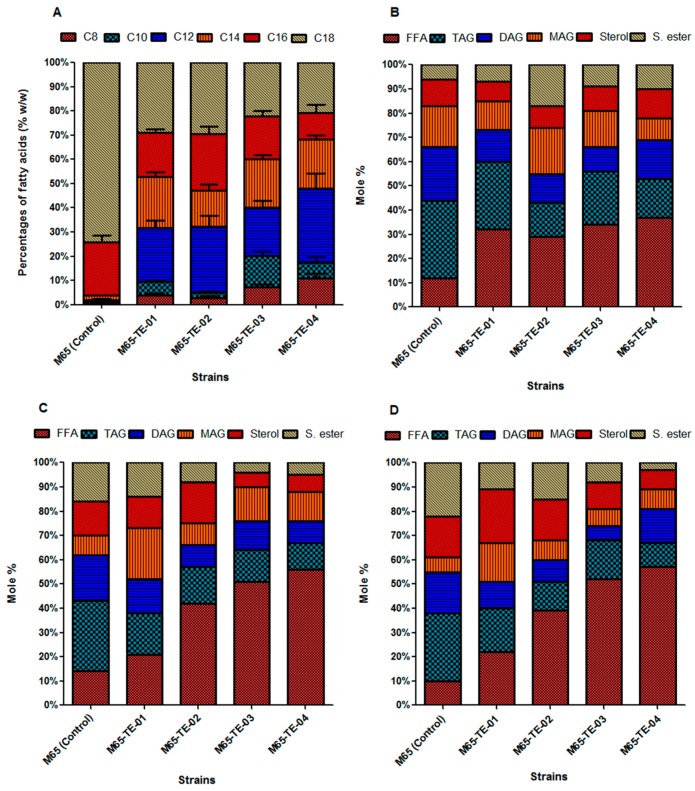
(**A**) Fatty Acid (FA) profiles of the control (M65) and the engineered strains, (**B**) Abundance of a medium-chain lipids, (**C**) 16-carbon lipids, and (**D**) 18-carbon lipids from *Mucor circinelloides* strains (M65) expressing different heterologous acyl-ACP thioesterase enzymes in different lipid classes, i.e., free fatty acids (FFA), triacylglycerol (TAG), diacylglycerol (DAG), monoacylglycerol (MAG), sterol and steryl ester (S. ester).

Oleaginous microorganisms mostly produced long chain fatty acids (LCFAs) (i.e., C16–C18). To generate significant amounts of MCFA, we heterologously expressed the acyl-ACP thioesterases (TEs) from bacterial and plant sources in *M. circinellodes* M65. This tactic revealed a substrate preference towards MCFA production [33,34]. Our results clearly showed that integration of diverse heterologous genes encoding acyl-ACP thioesterases proteins from *Anaerococcus tetradius*, *Cuphea palustris*, *Clostridium perfringens*, and *Umbellularia californica* in the recipient fungal strain (M65) switched the FAS complexes towards elevated production of MCFAs. Taken together, we concluded that the expression of each TE gene had the ability to produce more MCFAs (C8–C12) by terminating elongation of the fatty acyl-chain in distinctive amounts. The engineered strains generated maximum lipid production at 72 h, and the fatty acid composition was similar at different point times, thus the MCFA abundance was determined at that time. The engineered strains, i.e., M65-TE-01, M65-TE-02, M65-TE-03, and M65-TE-04, produced de novo fatty acids (C8 to C12) making up 30.52 ± 4%, 28.87 ± 3%, 39.3 ± 7%, and 46.55 ± 3%, respectively, of their total lipid contents with a yield ranging from 1.14 to 2.77 g/L (Figure 4A, Table 1). Conversely, the control strain produced very minute quantities of MCFA (i.e., 0.118 g/L) under the same growth conditions. Interestingly, we also demonstrated that the production of MCFAs was associated with depletion in LCFAs (i.e., C18) (Figure 4A).

### 2.5. Integration of De Novo Medium-Chain Fatty Acids (MCFAs) into Diverse Lipid Classes

To estimate the capability of strain *M. circinelloides* M65 to integrate the de novo MCFAs into different lipid classes (i.e., triacylglycerides (TAGs), diacylglycerides (DAGs), monoacylglycerides (MAGs), steryl ester, and sterols), we fractionated the total lipids into the aforesaid classes by chromatography, and the abundance of each fraction was calculated.

An inverse correlation was observed between the proportions of TAGs and free fatty acids (FFAs); likewise, the control strain contained a greater share of the TAG fraction (i.e., 32%) compared with the total lipid content in comparison to the engineered strains (i.e., 28%, 14%, 22%, and 16% for M65-TE-01, M65-TE-02, M65-TE-03, and M65-TE-04, respectively). Conversely, a greater abundance of FFAs was detected in all engineered strains, i.e., 32%, 29%, 34%, and 37% for M65-TE-01, M65-TE-02, M65-TE-03, and M65-TE-04, respectively) in comparison to the control strain (0.65%). Interestingly, these de novo MCFAs were found to integrate into all lipid classes. All fractions of total lipids for the engineered strains are depicted in Figure 4B. Our result showed that all engineered strains contained significant quantities of MCFAs in the TAG and FFA fractions (Figure 4B).

In addition to the inclusion of MCFAs into all lipid classes, the expression of heterologous acyl-ACP thioesterase from bacterial and plant sources also modified the localization of native fatty acids. For prospective C16 fatty acid production, the control strain contained only 0.45% fatty acids as FFAs, while the engineered strain showed an elevated trend in this aspect (i.e., 21%, 42%, 51%, and 56% for *TE-01*, *TE-02*, *TE-03*, and *TE-04*, respectively) (Figure 4C). Regarding C18 fatty acid production, an elevated trend for FFAs was also observed in the engineered strains in comparison to the control strain (Figure 4D).

## 3. Discussion

Oleaginous fungi are gaining attention due to their intrinsic ability to generate high titers of lipids from simple glucose molecules. Many investigators have genetically manipulated *M. circinelloides* to achieve elevated production of lipids and diverse chemical products [35,41,42,43,44]. In terms of the carbon chain length, *M. circinelloides* produces large quantities of long-chain fatty acids (C16–C18). Conversely, there is a greater demand for medium-chain fatty acids as substrates in many chemical, petroleum, pharmaceutical, and nutraceutical industries [1,2,3,4,5,6,7,8]. In the current investigation, diverse heterologous genes from bacterial and plants sources encoding acyl-thioesterase proteins were functionally expressed into *M. circinelloides* M65 to produce high titers of MCFAs. Moreover, the total lipid contents were also increased to levels ranging from 1.5 to 2.0-fold. This approach ultimately represented a potential tool to enhance the flux towards lipid accumulation pathways for augmented MCFA production in *M. circinelloides.*

The heterologously expressed acyl-thioesterases from bacterial and plant sources resulted in overproduction of MCFAs in the range from 30.52 to 46.55% of the total fatty acids. Amongst all mutant strains, the engineered strain, M65-TE-03 produced maximum abundance of decanoic acid (i.e., 20%), while octanoic acid was found at maximum level in strain M65-TE-04 (i.e., 10%). Moreover, the lauric acid was generated in significant amount in the all engineered strains (i.e., 10% to 45%). The engineered strains also presented an elevation of C16 and drastically decreased quantity of C18 fatty acids, and thus, based on the aforesaid discussion, we postulated that these de novo MCFAs were generated at the cost of 18-carbon fatty acids. The expression of acyl-ACP thioesterase protein from *Umbellularia californica* in the recipient fungus strain of *M. circinelloides* (i.e., M65-TE-04) resulted maximum generation of C8 and C12 (i.e., 10.5 and 30.73%) and noteworthy generation of C10 fatty acids (i.e., 7.1%). Moreover, the quantity of LCFAs (i.e., C16) was also increased 3-fold in this engineered strain. As discussed above, the increases in all C8 and C16 fatty acids were associated with a decline in the abundance of C18 lipids. The results of our current investigation revealed that acyl-ACP thioesterases exhibiting a substrate preference for medium-chain acyl-ACPs were integrated into the native fungal FAS reaction center and facilitated the earlier discontinuation of fatty acid elongation to eventually generate greater quantities of MCFAs. Our results are more significant than those of prior studies, which demonstrated that all tested thioesterases produced low titers of C8 and C10-containing lipids in *E. coli* and *Y. lipolytica* [13,17,32,45,46,47]. The mechanism by which acyl-ACP thioesterase from bacterial or plant sources integrates into the native fungal FAS system remains unclear, because fungal FAS exhibits a discrete configuration and specific mechanism for the termination step in the fatty acyl chain as compared to the prokaryotic and mammalian counterparts [48,49].

One possible mechanism is that the heterologous thioesterase (TEs) would localize in the reaction center of fungal FAS to facilitate the access of acyl-ACP/acyl-CoA substrates to their catalytic sites. This disruption might also indicate why the engineered strains showed reduced growth because of the FAS functioning incorrectly. It is noteworthy to mention that the rate of change in the growth pattern was also associated with the color variation of mycelia. This phenomenon might be due to the generation of MCFAs or altered physiology of the cell. A significant variation in color has been observed between the control and engineered strain, as depicted in Figure S1. In comparison to previous reports on *E. coli* and *Y. lipolytica*, our genetically manipulated strains produced high titers of MCFAs (C8–C12) (30.52–46.55%), representing the optimum integration of heterologous thioesterase with fungal FAS complexes. Furthermore, more noteworthy results were obtained when utilizing thioesterases from *Clostridium perfringens* and *Umbellularia californica* (i.e., M65-TE-03 and M65-TE-04, respectively). Despite the elevation in non-native fatty acid production, we also noticed a significant increase in total lipid titers during the process of fermentation. One possible reason for this observed increment in the engineered strains was primarily attributed to the specific function of TEs in reducing the feedback inhibition of several pathways, resulting in an elevated flux towards lipid production [50,51]. This aforesaid mechanism is not speculated in the C8 high-producing engineered strain (M65-TE-04) since it generated higher titers of LCFAs.

In nature, fatty acids are found in diverse classes of lipids, i.e., TAGs, DAGs, MAGs, sterol, steryl esters, and FFAs. This division necessitates the activity of various enzymes with specific activity towards de novo fatty acids (i.e., MCFAs). In our investigation, we noticed the presence of de novo fatty acids in all the aforementioned classes of lipids; thus, represents that FAS machinery of *M. circinelloides* M65 has the capability to permit the identification and alterations in fatty acids as short as C8. In comparison to the control strain, most engineered strains demonstrated different quantities of various lipid classes. In general, an inverse correlation was observed among FFAs, TAGs, and sterols, i.e., abundant FFAs were augmented in all engineered strains with a subsequent decline in TAG and sterol abundance. However, in the context of MCFAs, a similar trend was not observed, excluding a change in the LCFA length. The elevated amounts of non-native MCFAs were attributed to increments in the quantity of FFAs in all engineered strains.

## 4. Materials and Methods

### 4.1. Fungal Strains, Cultivation, and Transformation Conditions

The genomes of *Anaerococcus tetradius* (ATCC 35098)*, Cuphea palustris* (tx.ID 43077), *Clostridium perfringens* (JGS 1495), and *Umbellularia californica* (tx.ID 3438) were used as sources of acyl-ACP thioesterase (TE) genes. The uracil auxotroph strain M65, which has comparatively similar oil-producing/physiological characteristics to *M. circinelloides* WJ11 (GenBank accession No.: LGTF00000000) [36], was employed as a background strain for all transformation experiments to express the genes encoding the different thioesterase proteins. For all cloning experiments, *Escherichia coli* DH5α [52] was used and grown in Lysogeny broth (LB) medium at 37 °C with shaking at 220 rpm. Plasmid pMAT1552-PyrF [36] was used as the cloning as well as the expression vector. The cultures were subjected to growth at 26 °C in minimal media with casamino acids (MMC) or yeast peptone glucose (YPG) solid medium [53,54]. The aforesaid medium was supplemented with uridine at a rate of 200μg/mL as required. The pH was maintained at 4.5 for mycelia and 3.0 for colonial growth. Transformation was performed by the electroporation-mediated procedure as previously described by Torres-Martínez et al. [55]. Spores of *M. circinelloides* are multinucleate, so vegetative selection was mandatory to isolate homokaryotic transformants. The preliminary heterokaryotic transformants were subjected to vegetative rounds of growth on MMC medium to produce homokaryotic transformants [56]. The engineered strains M65-TE-01 (*TE-01* over-expressing strain), M65-TE-02 (*TE-02* over-expressing strain), M65-TE-03 (*TE-03* over-expressing strain), M65-TE-04 (*TE-04* over-expressing strain), and M65 (strain harboring the vector pMAT1552-pyrf, as a control strain) were initially cultivated with 100 μL of the spore suspension (~10^7^ spores/mL) in 500-mL flasks containing 150 mL of K and R medium [57]. Subsequently, these flasks were equipped with baffles to improve aeration and eventually incubated for 24h in a shaker at 150 rpm and 28 °C. The resultant seed culture was used for inoculation at 10% (*v*/*v*) into a 3-L fermenter (BioFlo/CelliGen 115, New Brunswick Scientific, Edison, New Jersey, NJ, USA) containing 1.5L of modified K and R medium (i.e., 80.0 g glucose/L). These fermenters were operated at 28 °C and stirred at 700 rpm with aeration at 0.5 vvm. The pH of the culture medium was constantly adjusted to 6.0 through the automated addition of 2M NaOH or 2M H_2_SO_4_ solutions. The culture samples of control and engineered strains were collected for analysis at 12, 24, 36, 48, 60, 72, 84, and 96 h based on the lipid accumulation characteristics.

### 4.2. Plasmids Construction

Four different acyl-ACP thioesterase (TE) genes were cloned into the pMAT1552-PyrF vector for expression in *M. circinelloides* M65 (i.e., a uracil auxotroph of *M. circinelloides* WJ11). This vector contained the *pyrF* gene of *M. circinelloides*, which was flanked up and down-stream by 1 kb of *carRP-carRP* sequences, and was subsequently employed for the development of the TE-over-expressing plasmid. The *pyrF* gene encodes uridine as a selectable marker, and flanking sequences corresponding to regions surrounding the carotenogenic *carRP-carRP* gene allow its chromosomal integration via homologous recombination (HR). The acyl-ACP thioesterase gene was isolated by PCR amplification from the genome of *Anaerococcus tetradius* (GenBank accession No.: KR180390, gene size: 699 bp), *Cuphea palustris* (GenBank accession No.: KR180392), gene size: 1239 bp), *Clostridium perfringens* (GenBank accession No.: KR180393, gene size: 744 bp), *Umbellularia californica* (GenBank accession No.: KR180394, gene size: 1152 bp) using primers pairs TE-01-F and TE-1R, TE-02-F and TE-02-R, TE-03-F and TE-03-R, and TE-04-F and TE-04-R, respectively (Supplementary materials: Table S1). The aforesaid primers exhibited homologous sequences (i.e., 28 bp) to the *Xho*I restriction sites in plasmid pMAT1552-pyrF, and the PCR fragment was finally cloned into plasmid pMAT1552-pyrF using the restriction endonuclease *Xho*I to construct the recombinant plasmids i.e., pMAT1552-pyrF-TE-01, pMAT1552-pyrF-TE-02, pMAT1552-pyrF-TE-03, pMAT1552-pyrF-TE-04 (One-Step Cloning Kit from Takara Bio USA, Inc., CA, USA). All primers used for plasmids construction and genes conformation are listed in Supplementary materials: Table S2.

### 4.3. Preparation for Genomic DNA and Quantitative Reverse Transcription Polymerase Chain Reaction (qRT-PCR) Analysis

To extract the genomic DNA, the over-expressing transformants (i.e., M65-TE-01, M65-TE-02, M65-TE-03, and M65-TE-04) of *M. circinelloides* were grown in K and R medium for 3 days at 28 °C with shaking (i.e., 150 rpm). Subsequently, the mycelia were harvested using the suction filtration protocol and washed three times with distilled water. Finally, the genomic DNA was extracted using the DNA quick Plant System Kit (Tiangen Biotech Beijing, Co., Ltd., Beijing, China) per the manufacturer’s instructions. PCR amplification with specific primers (i.e., pMAT1552F/R) was carried out to confirm whether different TE proteins (i.e., *TE-01*, *TE-02*, *TE-03*, and *TE-04*) had integrated into the genome of *M. circinelloides* (Supplementary materials: Table S2).

For quantitative reverse transcription PCR (qRT-PCR) analysis, engineered strains (i.e., M65-TE-01, M65-TE-02, M65-TE-03, and M65-TE-04) were grown in a 3-L fermenter with modified K and R medium, and the mycelia were harvested at intervals of 24, 48, and 72 h. Total RNA was isolated from the *M. circinelloides* strains using TRIzol after grinding in liquid N_2_, and reverse-transcribed using the Prime ScriptRT reagent kit (Takara Bio USA, Inc., CA, USA) according to the manufacturer’s instructions. qRT-PCR was performed using specific primers (Supplementary materials: Table S3) with the CFX Connect Real-Time System (Bio-Rad, CA, USA) and iTaq Universal SYBR Green PCR Supermix (Bio-Rad, CA, USA) according to the manufacturer’s instructions. The transcription of all TE genes was normalized to the levels of 18S rRNA mRNA, and the outcomes were elaborated as relative expression levels. The data were computed by the 2^−ΔΔ*C*t^ method.

### 4.4. Determination of the Dry Cell Weight (DCW) and Lipid Accumulation

Harvesting of the biomass was carried out by the suction filtration method using weighted filter paper, followed by three washes with distilled water to remove possible remaining medium, sequential freezing at −80 °C overnight and lyophilization. The weight of the biomass was calculated by the gravimetrical method. Lipid extraction was carried out as previously described by Folch et al. [58] with minor alterations. Approximately 15–20 mg of freeze-dried biomass was mixed with chloroform/methanol (2:1, *v*/*v*). Pentadecanoic acid (15:0 from Millipore Sigma, MO, USA) was added to the lyophilized cells as an internal standard. Methylation was performed with 10% (*v*/*v*) methanol anhydrous/methanolic HCl for 4 h at 60 °C [58]. Fatty acid methyl esters (FAMEs) were finally extracted with n-hexane and analyzed by GC with a DB-Waxetr column (30 m × 0.32 mm; film thickness, 0.25 m; Shimadzu Co., Ltd., Kyoto, Japan). The specifications used for the GC machine were as follows: 20 °C for 3 min, ramp to 200 °C at 5 °C min^−1^, ramp to 220 °C at 4 °C min^−1^, hold for 2 min [37].

### 4.5. Determination of Glucose and Nitrogen Contents in the Culture Medium

Glucose oxidase Perid-test kit (Shanghai Rongsheng Biotech Co., Ltd., Shanghai, China) was used to measure the glucose concentration in the culture, while the indophenol method was used according to Chaney and Marbach [59] to assay the ammonium concentration.

### 4.6. Separation of Lipid Classes

To calculate the abundance of various lipid classes in the engineered strains, total lipids were fractioned into their principle components. Hydrated florisil (7%) obtained by shaking overnight at room temperature was eventually used as an adsorbent for the chromatography procedure. Fractions were obtained from total lipid eluted according to the method described by Carroll [60], and the abundance of different eluted lipid classes was estimated according to the method described by Freeman and West [61].

### 4.7. Statistical Analysis

A statistical analysis of the obtained data was conducted using SPSS 16.0 for Windows (SPSS Inc., Chicago, IL, USA). The mean value and standard error of the mean were calculated from the data obtained from three independent experiments. Differences between means were measured by the Student’s *t* test, and *p* < 0.05 was regarded as significantly different.

## 5. Conclusions

In the present investigation, we found that four different TEs from bacterial and plant sources led to significant increments in lipid accumulation and evident changes in the fatty acid profile when heterologously over-expressed in *M circinelloides* M65. The elevated generation of MCFAs was accompanied by a concurrent decrease in native LCFA (i.e., C18) production, which indicated that the embedded TEs interfered with the fatty acid biosynthesis machinery (FASs) by channeling acyl-ACP molecules toward the non-native production of MCFAs. Finally, we also observed MCFAs integrated in all diverse lipid classes. Therefore, we concluded that *M. circinelloides* M65 can be engineered to produce MCFAs with improved metabolic flux through the FASs pathway.

## Figures and Tables

**Table 1:** Fatty acid composition in control and over-expressing strains of *M. circinelloides* M65.

	M65 (Control)	M65-TE-01	M65-TE-02	M65-TE-03	M65-TE-04
DCW (g/L)	13.25 ± 0.11	11.34 ± 0.31	06.12 ± 1.12	11.54 ± 0.82	09.14 ± 0.45
% TLC (DCW)	38.31 ± 0.19	65.32 ± 0.43	64.80 ± 1.91	57.01 ± 1.23	65.23 ± 1.42
TLC (g/L)	05.07 ± 0.11	07.40 ± 0.09	03.96 ± 0.07	06.57 ± 0.04	05.96 ± 0.73
% MCFA	02.33 ± 0.91	30.52 ± 1.14	28.87 ± 1.34	39.37 ± 0.79	46.55 ± 0.45
MCFA(g/L)	0.11 ± 0.04	02.25 ± 0.05	01.14 ± 0.02	02.58 ± 0.04	02.77 ± 0.02

Fatty acid portfolio of the control (M65) and engineered strains (i.e., M65-TE-01, M65-TE-02, M65-TE-03, and M65-TE-04) of *M. circinelloides* (M65). For each strain, total lipid (TLC) and MCFA contents are demonstrated in % *w*/*w* of DCW and % *w*/*w* of total lipid (g/L), respectively. The abundance of MCFA (C8-C12) contents is also demonstrated in g/L. Average and standard errors are provided for two clones cultivated separately. DCW and FA content were measured after 72 h of cultivation in modified K and R medium.

## Supplementary Materials

Supplementary materials can be found at http://www.mdpi.com/1422-0067/20/3/786/s1.

## Abbreviations

FAS	fatty acid synthase
MCFAs	medium-chain fatty acids
LCFAs	long chain fatty acids
DCW	dry cell weight
M.C	*Mucor circinelloides*
FFA	free fatty acid
SCO	single cell oil
TEs	thioesterases
TAG	triacylglycerol
DAG	diacylglycerol
MAG	monoacylglycerol
Tx	taxonomy
GC	gas chromatography
FAMEs	fatty acid methyl esters
