# Supplementary material for: Engineering of Fatty Acid Synthases (FASs) to Boost the Production of Medium-Chain Fatty Acids (MCFAs) in Mucor circinelloides
Source: Int J Mol Sci. 2019 Feb 12;20(3):786. doi: 10.3390/ijms20030786 (PMC6387429; doi:10.3390/ijms20030786)
Supplement: Supplementary file 1 [file ijms-20-00786-s001.pdf]

Engineering of Fatty Acid Synthases (FASs) to Boost the Production of Medium-Chain Fatty Acids (MCFAs) in *Mucor circinelloides*

Syed Ammar Hussain [1, 2, †], Ahsan Hameed [1, †], Md. Ahsanul Kabir Khan [1], Yao Zhang [1], Huaiyuan Zhang [1], Victoriano Garre [3] and Yuanda Song [1] *

[1] Colin Ratledge Center for Microbial Lipids, School of Agriculture Engineering and Food Science, Shandong University of Technology, 255049 Zibo, China

[2] Department of Biology, South Texas Center of Emerging Infectious Diseases (STCEID), University of Texas, 78249 San Antonio, USA

[3] Departamento de Genética y Microbiología (Unidadasociada al IQFR-CSIC), Facultad de Biología, Universidad de Murcia, 30071 Murcia, Spain

Author's information

[1, 2] **Syed Ammar Hussain**, PhD. Colin Ratledge Center for Microbial Lipids, School of Agriculture Engineering and Food Science, Shandong University of Technology, Zibo, P.R. China. Tel: +86-13287093112. Email: ammarshah88@yahoo.com

[1]**Ahsan Hameed**, PhD. Colin Ratledge Center for Microbial Lipids, School of Agriculture Engineering and Food Science, Shandong University of Technology, Zibo, P.R. China. Tel: +86-13287090124. Fax: +86-533-278094. Email: ahsanhameed@outlook.com

[1]**Md. Ahsanul Kabir Khan,** PhD. Colin Ratledge Center for Microbial Lipids, School of Agriculture Engineering and Food Science, Shandong University of Technology, Zibo, P.R. China. Tel: +86-13287096578. Email: kabir_khan@sdut.edu.cn

[1]**Yao Zhang**, PhD. Colin Ratledge Center for Microbial Lipids, School of Agriculture Engineering and Food Science, Shandong University of Technology, Zibo, P.R. China. Tel: +86-13287093112. Email: zhangyao@sdut.edu.cn

[1]**Huaiyuan Zhang**, PhD. Colin Ratledge Center for Microbial Lipids, School of Agriculture Engineering and Food Science, Shandong University of Technology, Zibo, P.R. China. Tel: +86-13287093112. Email: zhyuan004@126.com

[3] **Victoriano Garre**, PhD. Departamento de Genética y Microbiología (Unidad asociada al IQFR-CSIC), Facultad de Biología, Universidad de Murcia, 30071 Murcia, Spain.
Email: vgarre@um.es

[1]***Yuanda Song**, PhD (Professor & director). Colin Ratledge Center for Microbial Lipids, School of Agriculture Engineering and Food Science, Shandong University of Technology, Zibo, P.R. China, Tel: +86-139-06174047, Fax: +86-533-2780944. Email: ysong@sdut.edu.cn

Table S1 Primers for gene cloning

**TE-01-F**	ACTTTTATATACAAAATAACTAAATCTCGAGATGAAGTTCAAGAAGAAG
**TE-01-R**	ACTAGTCGCAATTGCCGCGGCTCGAGTTATTAGACGTTGGTCTTG
**TE-02-F**	ACTTTTATATACAAAATAACTAAATCTCGAGATGGTCGCTGCTGCTGCTTCTT
**TE-02-R**	ACTAGTCGCAATTGCCGCGGCTCGAGTTATTAGGTCTTACCGGTGGAAAT
**TE-03-F**	ACTTTTATATACAAAATAACTAAATCTCGAGATGGGTAAAGCTTACGAA
**TE-03-R**	ACTAGTCGCAATTGCCGCGGCTCGAGTTATTAGTACCAGTAGGTCTCAG
**TE-04-F**	ACTTTTATATACAAAATAACTAAATCTCGAGATGGCTACCACCTCTTTGGC
**TE-04-R**	ACTAGTCGCAATTGCCGCGGCTCGAGTTATTAGACACGAGGCTCA
**C-F**	GAAGGCTTTACTGTTCCTATCA
**C-R**	TCTCATTTTTCCCTGTCTGC

Table S2 Primers for gene confirmation

**pMAT1552-F**	GAAGGCTTTACTGTTCCTATCA
**pMAT1552-R**	GACAGCAAGTGAGGAGAGGAACAGC

Table S3 Primers for RT-qPCR analysis

**RT-1F**	ATACCTGGTCGCTCTCATGAAC
**RT-1R**	CCTCGTGACTCAACGATAAAGT

**RT-2F**	ACTGGTCTTCCTCTCTCTCTCC
**RT-2R**	GGGAGGAGGACAAAGTATCTTC
**RT-3F**	CCGATAATTCTTCTGAGACCAC
**RT-3R**	GTCCATATCGTAGGCTTCAAAC
**RT-4F**	GCTAAGTCTGTCGGTATCTTGG
**RT-4R**	ATACCGTTGTTACCAGAAGCAC

Figure S1. (**A**-**E**) Color variation among dried mycelia of engineered, and control strain. (**A**) Control strain, (**B**) M65-TE-01, (**C**) M65-TE-02, (**D**) M65-TE-03, (**E**) M65-TE-04 strain.

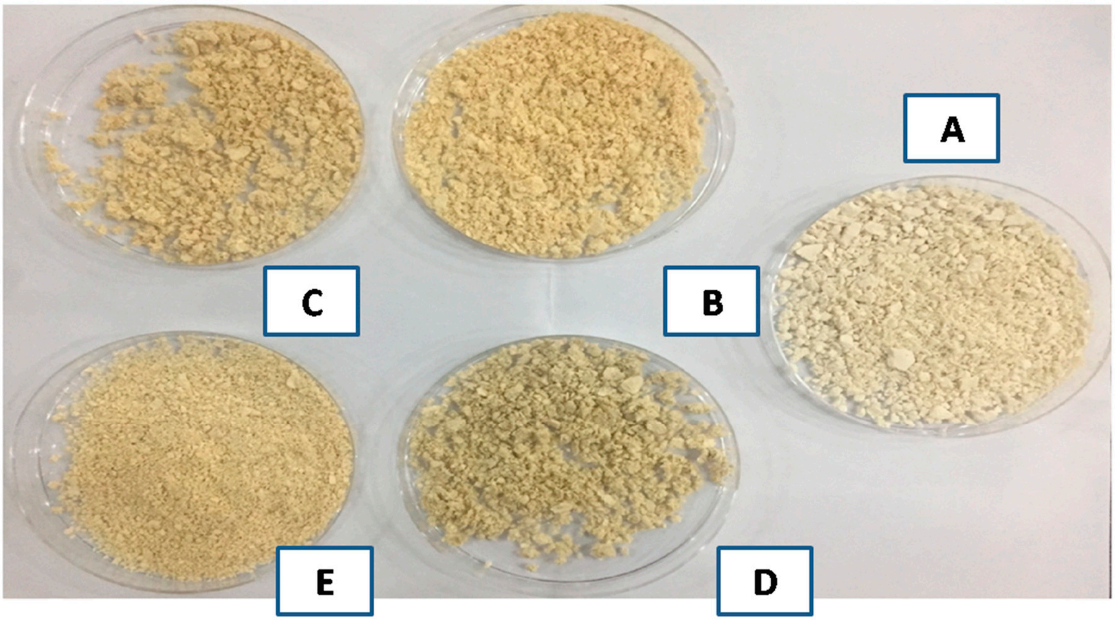

Funding: This work was supported by Chinese Government Scholarship Council (CSC), National Natural Science Foundation of China (31670064), TaiShan Industrial Experts Program (tscy 20160101), and starting grant from Shandong University of Technology.

Acknowledgments: We thank to Wu Yang and Junhaun Yang for technical support. We are also indebted to Scarlett Geunes-Boyer, Yusuf Nazir, and Alexis Garcia for critical reading and discussions.

Conflict of Interest: Authors declare no competing interests.

Note: Syed Ammar Hussain & Ahsan Hameed will be considered equally first authors for this manuscript.
